# Spectroscopic Identification of Carbamate Formation and Synergistic Binding in Amide–CO_2_® Complexes

**DOI:** 10.1021/acs.jpclett.5c03351

**Published:** 2025-12-08

**Authors:** Jia Han, Jiaye Jin, Hannah Buttkus, Anne B. McCoy, Knut R. Asmis, Timothy S. Zwier

**Affiliations:** † Wilhelm-Ostwald-Institut für Physikalische und Theoretische Chemie, 9180 Universität Leipzig, Linnéstraße 2, 04103 Leipzig, Germany; ‡ Department of Chemistry, 7284 University of Washington, Seattle, Washington 98195, United States; § Gas Phase Chemical Physics, Sandia National Laboratories, Livermore, California 94550, United States

## Abstract

Carbamate formation is an elementary step that governs nitrogen-centered nucleophilic CO_2_ capture across diverse environments, yet a direct, structure-specific understanding has been lacking. Here, we report the first gas-phase characterization of closed-shell carbamate formation by deprotonated amides, using cryogenic ion trap vibrational spectroscopy combined with quantum chemical calculations. Reactions of deprotonated benzamide and isophthalamide anions with CO_2_ form carbamate species, with the amide nitrogen serving as the nucleophilic site. Diagnostic, strongly red-shifted antisymmetric CO_2_ stretching vibrational bands, supported by a bonding analysis, establish chemisorption with substantial charge transfer. In the multiamide system, an intramolecular N–H···O hydrogen bond provides synergistic stabilization, correlating with larger red shifts and more exothermic binding. These structure-assigned benchmarks provide molecular-level insights into the binding motif, charge redistribution, and hydrogen bond-mediated stabilization of amide carbamates, which aid the characterization of carbamate formation in condensed phase.

## Graphical abstract

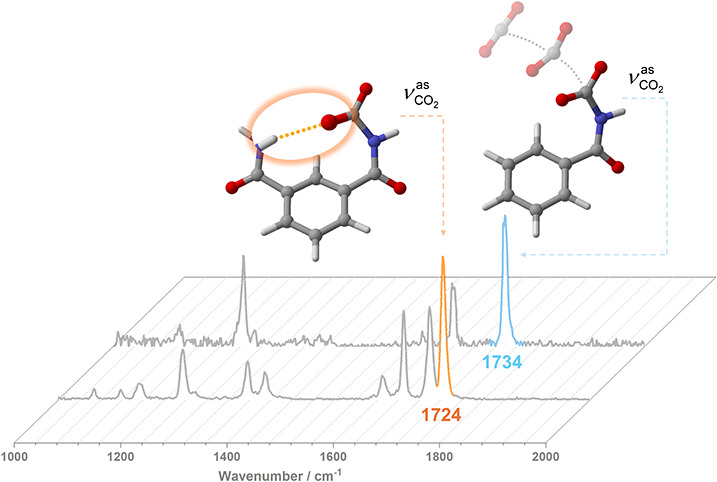

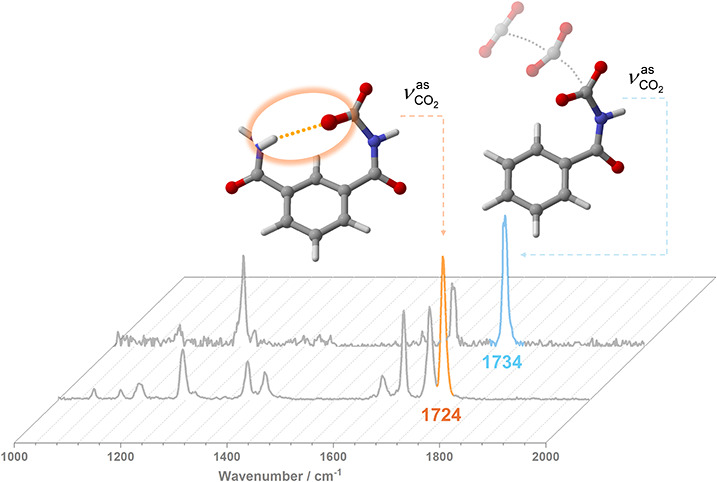

Rising anthropogenic CO_2_ emissions threaten the climate and ecosystems, motivating the development of CO_2_ capture technologies to mitigate their environmental impact.^1,2^ Direct air capture (DAC) offers the potential to eliminate past emissions and regulate atmospheric CO_2_ concentration with great flexibility.^3–6^ Efficient CO_2_ capture at such a low atmospheric concentration (0.04%) requires sorbent materials with both favorable and highly selective CO_2_ binding energetics. Solid sorbents functionalized with amine groups on porous silica-based supports, organic polymers, or metal-organic frameworks, have emerged as a leading strategy for DAC owing to their remarkable efficiency and selectivity.^7–10^ This performance arises not only from strong chemical interactions between CO_2_ and amine groups, but also from stabilizing hydrogen-bonding networks.^9,11–13^ The capture mechanism involves nucleophilic attack of the amine nitrogen on the electrophilic carbon of CO_2_ and proton transfer, ultimately yielding carbamate species as the primary chemisorption product (Supporting Information Scheme S1) whose persistence also depends sensitively on the local environment.^14–18^ A thorough understanding of carbamate formation and its stability is therefore essential, as it provides fundamental insights that are highly valuable for elucidating the more complex behavior of nucleophilic materials in CO_2_ capture.

While primary and secondary alkylamines are principally used in CO_2_ capture, from a fundamental standpoint, it is worthwhile to consider carbamate formation in other R–NH and R–NH_2_ groups. Primary amides, R–(C=O)–NH_2_, replace oxidatively labile CH_2_ units near reactive NH_2_ sites with C=O groups (Scheme 1). While the electron-withdrawing nature of the carbonyl group reduces nitrogen basicity and thus weakens CO_2_ binding, its NH_2_ hydrogen atoms are more acidic, making it possible to generate the carbamate anions under conditions where amines would not (see Supporting Information for details). To assess this possibility, it is important to understand the binding energetics and structural configurations of the amide-based carbamate species formed upon CO_2_ adsorption at amide nitrogen atoms.

**Scheme 1:**
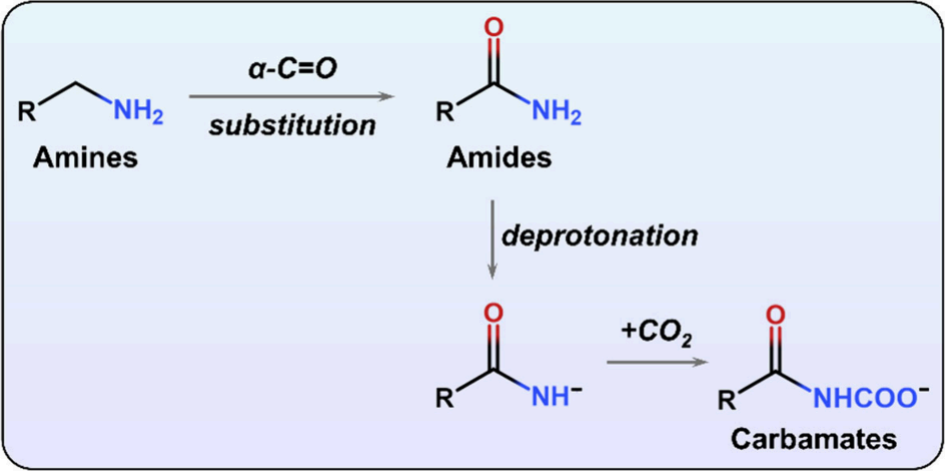
Structural Difference between Amine and Amide Species upon *α*-Carbon Substitution and the Formation of Carbamates through the Reaction of Deprotonated Amides with CO_2_

Although carbamates have been observed in condensed-phase amine systems, their unambiguous characterization is often obscured by the presence of multiple interacting components.^19–21^ Investigations of gas-phase cluster systems are free from such complications, in contrast, and have proven useful in providing molecular level insights. In particular, vibrational action spectroscopy of mass-selected complexes affords structure-specific characterization, while enabling detailed dissection of individual intermolecular interactions.^22–32^ Previous vibrational predissociation and photoelectron spectroscopic studies on anionic complexes between CO_2_ and N-heterocycles including pyridine, quinoline and azabenzenes, generated by low-energy electron attachment, confirmed carbamate formation in the presence of an excess electron.^33–36^ Herein, we employ infrared photodissociation (IRPD) spectroscopy in conjunction with high-level electronic structure calculations to investigate the reaction of CO_2_ with deprotonated benzamide (BZA) and isophthalamide (IPA). The results present the first spectroscopic evidence of CO_2_ chemisorption via electronically closed-shell carbamate formation in the gas phase.^33^ Moreover, introducing both nucleophilic and electrophilic sites into multiamide frameworks reveals synergistic intramolecular hydrogen-bond stabilization. These gas-phase, structure-assigned benchmarks aid the interpretation of carbamate-related vibrational bands and interaction trends in more complex environments.

Benzamide, a prototypical aromatic amide bearing a single −C­(=O)­NH_2_ group attached to the phenyl ring, was chosen as the first model system. Its structural simplicity isolates intrinsic amide–CO_2_ interactions and facilitates IR spectroscopic characterization in the fingerprint region, thus providing a reliable benchmark for comparison with theoretical predictions. Notably, its amine analogue benzylamine exhibits a relatively fast CO_2_ uptake rate, high cyclic capacity and reduced equipment corrosion,^37–39^ making CO_2_ capture by the corresponding amide an instructive subject of investigation.

To probe the vibrational signature of the CO_2_-adsorbed carbamate species, the [BZA_–H_(CO_2_)]^−^ complex was generated through the reaction between the deprotonated benzamide anion (BZA_–H_^–^) and CO_2_ in the gas phase. The BZA_–H_^–^ anion was obtained by proton abstraction at the NH_2_ group by fluoride anions (F^–^), whose high proton affinity drives the deprotonation process (Table S3). This deprotonation process likely occurs within the nanospray region and/or ion guides; that is, under gas-phase conditions. BZA_–H_^–^ was introduced into the gas phase using a custom-built nanospray ion source interfaced to a cryogenic ion trap triple mass spectrometer, as described previously.^40^ After generation, the gaseous anions were transferred into a He-buffer-gas-filled radiofrequency (RF) ion guide, where they encountered a steady flow of CO_2_ in a second RF ion guide, leading to formation of the adduct between BZA_–H_^–^ and CO_2_ through stabilizing collisions with the carrier gas. A vibrational action spectrum of the resulting anion complex was obtained by measuring the IRPD spectrum of the corresponding D_2_-tagged species, formed in the cryogenic ion trap, as presented in the top panel of Figure 1. From inspection of the spectral region below 2400 cm^–1^, it is directly apparent that the characteristic antisymmetric stretching band of bare CO_2_ at 2349 cm^–1^, typically expected for intact, weakly physisorbed CO_2_, is absent from this spectrum.^41^

**Figure 1:**
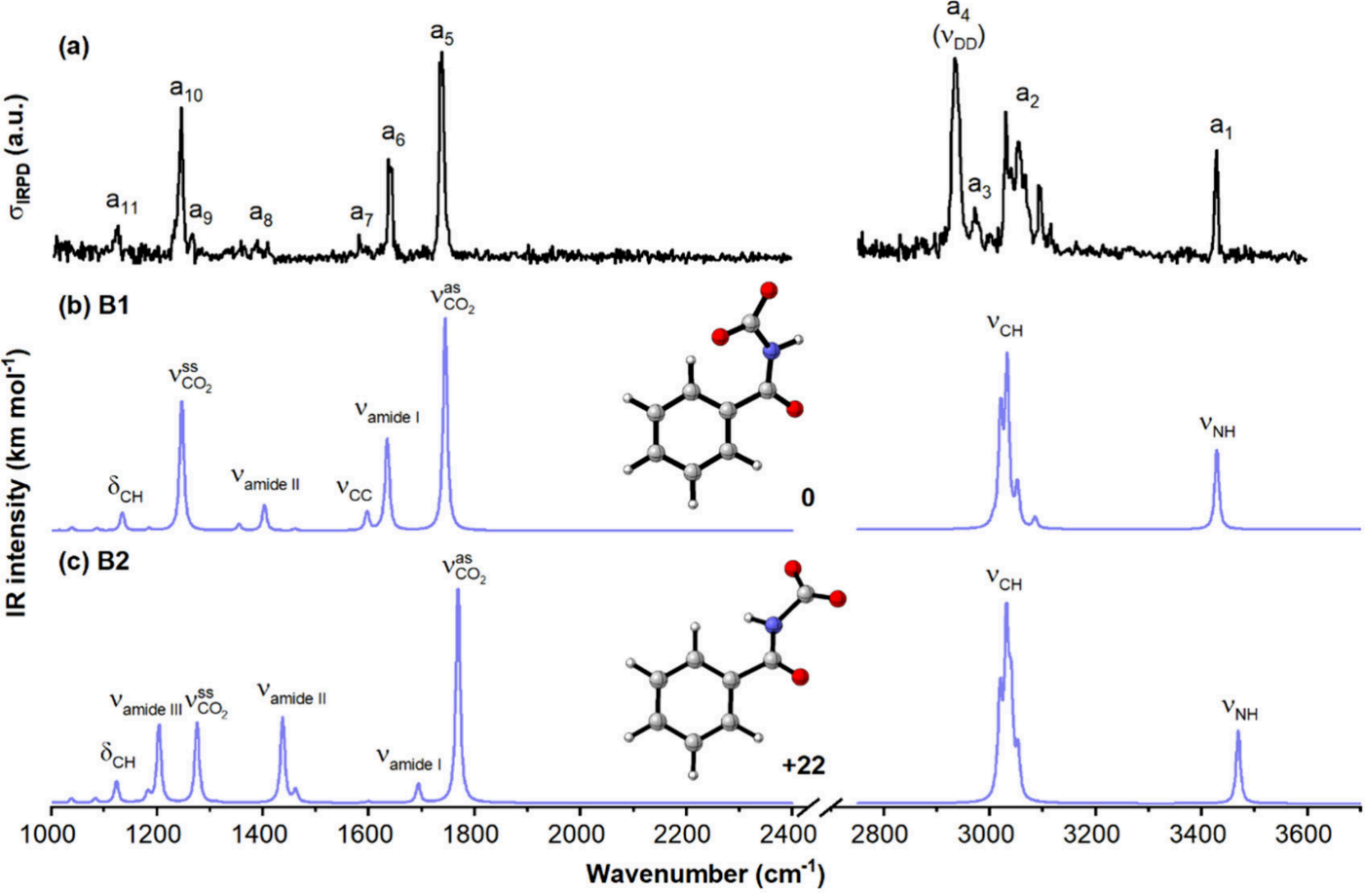
Comparison of the IRPD spectrum of the D_2_-tagged [BZA_–H_(CO_2_)]^−^ anion (a) with calculated harmonic spectra for two low-energy isomers **B1** and **B2**, (b and c) using the B3LYP-D3BJ/aug-cc-pVTZ method. Their relative energies (in kJ mol^–1^) are shown next to the structures. Scaling factors of 0.988 and 0.956 were applied to the 1000–2400 and 2750–3700 cm^–1^ regions, respectively. Calculations that consider messenger as well as anharmonic effects are included in Figure S2 and Table S1 of the Supporting Information.

To assist in assigning the IRPD spectrum, electronic structure calculations were performed and linear absorption spectra for low-energy structures were derived from scaled harmonic vibrational frequencies and intensities. Two carbamate isomers were identified for [BZA_–H_(CO_2_)]^−^, denoted as **B1** and **B2**, which originate from two distinct configurations of BZA_–H_^–^ that differ in the −NH orientation (Figure S4). Upon CO_2_ binding, these configurations give rise to complexes in which the −NH group is oriented either away from (**B1**) or toward (**B2**) the phenyl ring. Consistent with the relative stability of BZA_–H_^–^ conformers, **B1** represents the global minimum on the [BZA_–H_(CO_2_)]^−^ potential energy surface, whereas **B2** is predicted to be higher in energy by 22 kJ mol^–1^ using the B3LYP-D3BJ/aug-cc-pVTZ method.^42–45^ The simulated IR spectrum of **B1** (Figure 1b) satisfactorily recovers the position and relative intensity of nearly all observed bands. In the lower-energy region, two strong IRPD bands at 1734 cm^–1^ (a_5_) and 1248 cm^–1^ (a_10_) correspond to the excitation of the antisymmetric ( $\nu_{{CO}_{2}}^{\mathrm{as}}$ ) and symmetric ( $\nu_{{CO}_{2}}^{ss}$ ) stretching modes of the bound CO_2_ moiety, respectively, giving a splitting Δν_as‑ss_ of 486 cm^–1^. This is comparable to that reported for the pyridine–CO_2_^–^ radical anion carbamate (457 cm^–1^),^33,36^ but significantly larger than for the carboxylate groups of benzoate (315 cm^–1^)^46^ and acetate (279 cm^–1^)^47^ anions. The larger splitting in carbamate relative to carboxylate originates partly from the unusually high frequency of the antisymmetric stretch fundamental $\nu_{{CO}_{2}}^{\mathrm{as}}$ in the carbamate due to the reduced anionic charge on the CO_2_ moiety resulting from its conjugation with the adjacent amide group. At the same time, the frequency of the symmetric stretch mode, $\nu_{{CO}_{2}}^{ss}$ , in the acyl carbamate is unusually low because it is less localized on the (CO_2_)^−^ group than in the carboxylates, mixing with the two N–C stretching displacements on either side of the amide nitrogen.

Peaks characteristic of the **B1** carbamate are also detected at 1640 cm^–1^ (a_6_), 1582 cm^–1^ (a_7_), 1388 cm^–1^ (a_8_), and 1128 cm^–1^ (a_11_), and are assigned to the amide I vibration, CC stretching of the benzene ring, amide II vibration, and CH bending mode, respectively. Detailed band positions and assignments are provided in Figure S2 and Table S1. In contrast, the match obtained for **B2** is significantly poorer, particularly for amide-related vibrations: its amide I and II vibrational modes of **B2** are predicted at 1693 and 1437 cm^–1^, respectively, with relative intensities opposite to those observed experimentally, while the predicted amide III vibrational transition at 1203 cm^–1^ has no corresponding feature present in the experimental spectrum.

The considerable red shift of $\nu_{{CO}_{2}}^{\mathrm{as}}$ by 615 cm^–1^ relative to that of free CO_2_ reflects substantial chemical bond formation and charge transfer in the complex. The electrophilic carbon atom is attracted to the negatively charged nitrogen site, facilitating electron donation from the amide group to the vacant π* orbital of CO_2_. The resulting excess electron density induces an elongation of the C=O bonds by 7 pm relative to that in CO_2_ neutral and a decrease in the OCO bond angle by almost 50° from the linear geometry (Figure S4). Accordingly, the observed red shift of $\nu_{{CO}_{2}}^{\mathrm{as}}$ serves as a sensitive probe for characterizing CO_2_ charge distribution.^48^ Although isolated CO_2_ has a negative electron affinity (−0.6 eV) and consequently (CO_2_)^−^ is unstable in the gas phase, matrix isolation infrared studies have determined its $\nu_{{CO}_{2}}^{\mathrm{as}}$ to lie near 1660 cm^–1^.^49–51^ In situ IR spectroscopic studies of CO_2_-loaded amine sorbents have assigned absorptions in the 1530–1580 cm^–1^ region to the antisymmetric stretching mode of the carbamate (CO_2_)^−^ moiety.^19–21^ The IRPD band at 1734 cm^–1^, though higher than condensed-phase values, nonetheless represents a characteristic spectroscopic signature of CO_2_ chemisorption accompanied by electron transfer. This finding provides strong evidence that deprotonated amide functionalities can capture CO_2_ in a manner analogous to amines through nucleophilic attack at the carbon atom of CO_2_, yielding carbamate species.

The assigned structure **B1** for [BZA_–H_(CO_2_)]^−^ is unusual in several respects. First, unlike the amine-based carbamate, **B1** is an *N*-acyl carbamate, with the (CO_2_)^−^ moiety bonded to a conjugated amide group rather than a secondary amine. Second, the lowest energy structure (**B1**) incorporates a *cis*-amide group rather than *trans*-amide (**B2**), even though secondary *cis*-amides are about 10 kJ mol^–1^ higher in energy than their *trans*-amide counterparts.^52^ Finally, structure **B1** has the acyl carbamate side chain oriented out-of-plane relative to the phenyl ring, despite the loss of conjugation with the ring that this entails. By reorienting out-of-plane, the carbamate side chain avoids steric effects between the (CO_2_)^−^ and the phenyl ring CH groups.

CO_2_ capture by amine-based sorbents via reactions with polyamine groups is well documented to be energetically favorable, with adsorption enthalpies typically ranging from −50 to −100 kJ mol^–1^ at room temperature.^7,53^ Among recent advances, a polyamine-functionalized covalent organic framework (COF) material has shown superior performance in DAC applications, with a calculated CO_2_ adsorption energy of −101 kJ mol^–1^.^54^ To gain preliminary insight into the CO_2_ affinity of amide-based materials, density functional theory (DFT) calculations were performed to evaluate the reaction energies associated with CO_2_ binding at deprotonated amide functional groups. For BZA_–H_^–^, the calculated binding energy and enthalpy at 298 K are −75 and −77 kJ mol^–1^, respectively, showing a moderate CO_2_ binding affinity.

Next, we examined the deprotonated isophthalamide anion (IPA_–H_^–^), which features an additional amide group positioned meta relative to the single amide group in BZA_–H_^–^ (Figure S5). To probe the influence of this extra binding site, IRPD spectroscopy was employed to characterize the binding motifs of the corresponding complex, [IPA_–H_(CO_2_)]^−^. This complex was generated in the gas phase through the reaction of with CO_2_, similar to the formation of [BZA_–H_(CO_2_)]^−^. Figure 2 (top panel) shows the experimental IRPD spectrum of the D_2_-tagged [IPA_–H_(CO_2_)]^−^ complex. The spectral region can also be divided into two parts: the region above 2750 cm^–1^, dominated by DD and XH (X = C, N) stretching fundamentals, and the region below 2400 cm^–1^, containing bands related to amide vibrations and modes of the attached CO_2_ unit. The most intense band in the second region is observed at 1724 cm^–1^ (b_8_), indicating the occurrence of significant CO_2_ chemisorption.

**Figure 2:**
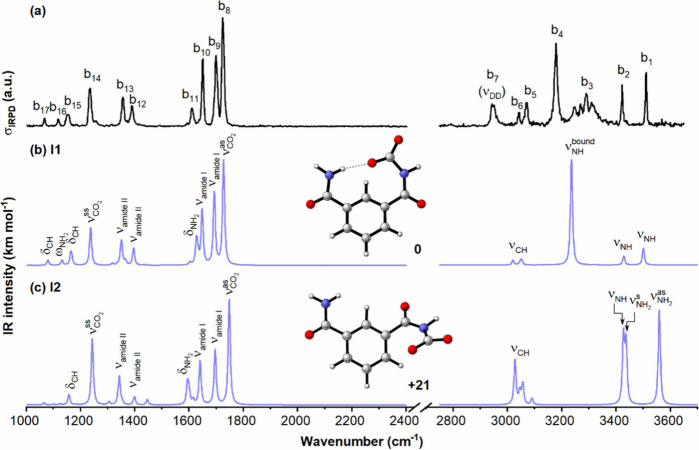
Comparison of the IRPD spectrum of D_2_-tagged [IPA_–H_(CO_2_)]^−^ (a) to calculated harmonic spectra for two low-energy isomers **I1** and **I2**, (b and c) using the B3LYP-D3BJ/aug-cc-pVTZ method. Their relative energies are shown next to the structures in kJ mol^–1^. Scaling factors of 0.988 and 0.956 were applied to the 1000–2400 and 2750–3700 cm^–1^ regions, respectively. Calculations that consider messenger as well as anharmonic effects are included in Figure S3 and Table S2.

To allow for an unambiguous structural assignment, the experimental IRPD spectrum of [IPA_–H_(CO_2_)]^−^·D_2_ is compared to simulated B3LYP-D3BJ/aug-cc-pVTZ IR spectra of the two most stable structures (Figure 2b,c). The two isomers found for [IPA_–H_(CO_2_)]^−^ differ in the relative orientation of the two amide groups in IPA_–H_^–^, corresponding to the *syn* and *anti* arrangements of the bare anion (Figure S5). The two bare ions are nearly isoenergetic (Δ*E* = 0.2 kJ mol^–1^), indicating that both conformers are thermally accessible. CO_2_ binding to the *syn*-IPA_–H_^–^ favors the formation of an intramolecular N–H···O hydrogen bond with one of the CO_2_ oxygen atoms, yielding the **I1** carbamate structure. In contrast, CO_2_ attachment to *anti*-IPA_–H_^–^ does not allow such hydrogen-bond stabilization, resulting in the **I2** carbamate structure, which is 21 kJ mol^–1^ higher in energy than **I1**. The experimental spectrum is in excellent agreement with the simulated spectrum of the lowest-energy isomer **I1**, both with regard to band positions and relative intensities. In the low-frequency region, the observed bands at 1724 cm^–1^ (b_8_) and 1236 cm^–1^ (b_14_) can be attributed to $\nu_{{CO}_{2}}^{\mathrm{as}}$ and $\nu_{{CO}_{2}}^{ss}$ , respectively, while bands b_9–10_ and b_12–13_ are assigned to the amide I and II vibrational modes of the two amide groups (detailed band positions and assignments are provided in Figure S3 and Table S2). These spectral features are consistent with the picture established for [BZA_–H_(CO_2_)]^−^, as the red-shifted $\nu_{{CO}_{2}}^{\mathrm{as}}$ provides direct evidence for electron transfer from the amide group to the adsorbed CO_2_, which is further corroborated by the electrostatic potential surface analysis shown in Figure S6. Importantly, the larger red shift of 625 cm^–1^ observed here reflects stronger chemisorption relative to that of [BZA_–H_(CO_2_)]^−^. Consistent with this, the structure **I1** (Figure S5) has an OCO angle of 131°, slightly smaller than the corresponding angle in the **B1** complex (133°). The additional bending likely arises from increased excess electron density on the CO_2_ unit facilitated by the additional amide group, as will be further discussed below.

In the high-frequency region, we anticipate the presence of three NH stretch fundamentals due to the NH_2_ and amide NH groups in [IPA_–H_(CO_2_)]^−^. The experimental spectrum contains peaks at 3511 cm^–1^ (b_1_) and 3422 cm^–1^ (b_2_), corresponding to NH stretch excitation of the free NH of the NH_2_ group and the free amide NH, respectively. In order to form the intramolecular hydrogen bond, the carbamate’s NH group is in a *cis*-amide configuration, which lowers its frequency by about 50 cm^–1^ with respect to that of the free NH stretch of the *trans*-amide group (∼3470 cm^–1^).^55^ The most intense and broader feature at 3178 cm^–1^ (b_4_) is assigned to NH stretch fundamental of the hydrogen-bonded NH_2_ group, which is nearly a localized NH stretch due to formation of the bridging hydrogen bond. Its frequency is red-shifted by hydrogen-bond formation to an extent that correlates with the hydrogen-bond strength.^56^ This assignment is supported by a prediction for the anharmonic frequency of the bound NH fundamental at 3157 cm^–1^ (Figure S3). In addition, the closely spaced transitions that make up b_3_ arise from a combination of strong coupling between the NH stretching vibration and low-frequency motions and Fermi-resonance interactions that mix the NH (ν = 1) level with overtones and combination bands involving $\nu_{{CO}_{2}}^{\mathrm{as}}$ , the two amide I vibrations, and the NH_2_ bending mode.

Notably, the predicted spectrum for **I2** does not match the experimental spectrum, particularly in the NH stretching region. In the absence of the intramolecular NH···O=C hydrogen bond, the free NH_2_ group results in two characteristic IR bands, assigned to the antisymmetric and symmetric NH_2_ stretching bands, as well as the free NH stretch at 3427 cm^–1^, which are not observed in the experimental spectrum, further corroborating the assignment to **I1** as the dominant binding motif.

Comparison of the assigned structures for the two CO_2_-adsorbed amides reveals analogous carbamate formation. The predicted lengths of the newly formed N–C bond are 151 and 150 pm for **B1** and **I1**, respectively, slightly longer than for a typical N–C single bond (146 pm).^57^ This deviation is supported by Mayer bond order values of 0.89 for both complexes,^58^ reflecting a bonding interaction with a mixed covalent-ionic character that originates from partial delocalization of electron density from the amide nitrogen into the CO_2_ π* orbital. Localized molecular orbital (LMO) analysis further identifies two similar orbital contributions to the N–C bonding framework (Figure S7). In both cases, the primary contribution arises from σ-type orbitals, accounting for 89% in **B1** and 87% in **I1** of the N–C Mayer bond, while secondary π-type orbitals contribute 11% and 13%, respectively. This analysis indicates that CO_2_ chemisorption at the amide site through carbamate formation is dominated by formation of a single N–C bond via σ-donation with auxiliary π contributions.

As discussed above, for [IPA_–H_(CO_2_)]^–^ there is an additional N–H···O hydrogen bonding interaction between an oxygen atom of CO_2_ and the intact amide group in the **I1** complex.^59^ To maximize the strength of this hydrogen bond, the donating amide group internally rotates out of the plane of the phenyl ring, forming a hydrogen-bonded bridge between the amide and carbamate side chains over the top of the phenyl ring. This interaction accounts for the experimentally observed red shift of the NH stretch fundamental.

To evaluate the impact of the intramolecular hydrogen bond on the CO_2_ binding affinity of the amide group, DFT calculations were performed, showing that the reaction energy, Δ*E*, and the reaction enthalpy at 298 K, Δ*H*(298 K), for CO_2_ capture by IPA_–H_^–^ are more exothermic by 10 and 12 kJ mol^–1^, respectively, compared to BZA_–H_^–^, underscoring the stabilizing effect of this secondary interaction. To gain deeper insight into the higher CO_2_ binding affinity of IPA_–H_^–^, the N–H···O hydrogen bonding strength was quantified through analysis of the electron density (ρ) at its bond critical point (BCP), yielding ρ = 0.020 au; based on the reported linear correlation between ρ at the BCP and binding energies,^60^ this corresponds to a bond energy of 33 kJ mol^–1^.

Optimal CO_2_ affinity of sorbents is highly application dependent. For postcombustion capture from flue gas, relatively weak to moderate interactions are preferred to ensure facile regeneration at mild conditions, whereas stronger binding becomes advantageous under very low CO_2_ partial pressures relevant to DAC.^61,62^ However, excessively high CO_2_ affinity, as often encountered in conventional amine sorbents, leads to high regeneration costs and reduced durability due to their large binding enthalpies.^63^ These findings demonstrate how additional hydrogen-bonding interactions in multiamide or mixed amide–amine systems could modulate CO_2_ affinity, illustrating a molecular-level strategy for fine-tuning the binding energetics to balance capture efficiency with energy-efficient release.

It should be noted that efficient capture of CO_2_ from ambient air requires not only optimal CO_2_ binding ability but also high selectivity. Given the low concentration of CO_2_ (0.04%) and the large quantities of N_2_ (78%) and O_2_ (21%) in the atmosphere, selective CO_2_ capture is critical to ensure efficient separation and subsequent sequestration or utilization. DFT calculations (Figure S8) were carried out to assess the selectivity of IPA_–H_^–^, showing that its binding affinity for N_2_ and O_2_ is at least six times weaker than for CO_2_. This large difference indicates an intrinsic CO_2_ preference of deprotonated amide groups over the major background gases. Other binding partners such as H_2_O are also relevant, but outside the scope of the present work.

In summary, we have investigated carbamate formation upon CO_2_ capture by deprotonated amide anions, BZA_–H_^–^ and IPA_–H_^–^, using IRPD spectroscopy in combination with high-level DFT calculations. The spectral signatures allow unequivocal structural assignment of the gas-phase CO_2_-adsorbed carbamate complexes, establishing that the amide nitrogen serves as the nucleophilic site in close analogy to amine-based systems. Furthermore, the presence of additional electrophilic sites in multiamide frameworks promotes a synergistic binding mechanism, where hydrogen-bonding interactions between the oxygen atoms of the carbamate anion and nearby amide functionalities could further stabilize the carbamate and strengthen the interaction energy. These insights deepen the fundamental understanding of carbamate chemistry and suggest the promise of amide functionalities for DAC. Future work is required to validate their performance under realistic conditions, including cyclic stability, regeneration energetics, and humidity tolerance.

## Supplementary Material



## Notes

Methods (section S1), quadrupole mass spectra (section S2), band assignments (section S3), calculated structures (section S4), and other aspects of CO_2_ binding in amide vs amine carbamates (section S5) (PDF)

## ∥

Department of Chemistry, State Key Laboratory of Porous Materials for Separation and Conversion, Shanghai Key Laboratory of Molecular Catalysis and Innovative Materials, Fudan University, Songhu Rd. 2005, 200438 Shanghai, China
