# Supplementary material for: Spectroscopic Identification of Carbamate Formation and Synergistic Binding in Amide–CO2® Complexes
Source: J Phys Chem Lett. 2025 Dec 8;16(50):12813–9. doi: 10.1021/acs.jpclett.5c03351 (PMC12720224; doi:10.1021/acs.jpclett.5c03351)
Supplement: Supplementary file 1 [file jz5c03351_si_001.pdf]

Supporting Information for

Spectroscopic Identification of Carbamate Formation and Synergistic Binding in Amide-CO_2^- Complexes

Jia Han,[a] Jiaye Jin,[a, §] Hannah Buttkus,[a] Anne B. McCoy,[b] Knut R. Asmis,*[a] Timothy S. Zwier*[c]

[a] Wilhelm-Ostwald-Institut für Physikalische und Theoretische Chemie, Universität Leipzig, Linnéstraße 2, 04103 Leipzig, Germany

[b] Department of Chemistry, University of Washington, Seattle, Washington 98195, United States

[c] Gas Phase Chemical Physics, Sandia National Laboratories, Livermore, California 94550, United States

[§] Present address: Department of Chemistry, State Key Laboratory of Porous Materials for Separation and Conversion, Shanghai Key Laboratory of Molecular Catalysis and Innovative Materials, Fudan University, Songhu Rd. 2005, 200438 Shanghai, China

* Email: knut.asmis@uni-leipzig.de; tszwier@sandia.gov

Table of Contents

S1. Methods S3

S1.1 Experimental methods S3

S1.2 Theoretical methods S4

S2. Quadrupole mass spectra S5

S3. Band assignments S6

S4. Calculated structures S10

S5. Other aspects of CO_2 binding in amide vs amine carbamates S12

S5.1 Reaction mechanisms of amine systems with CO_2 ....S12

S5.2 Proton affinities of F^-, BZA_{-H}^-, IPA_{-H}^- , and $BZAN_{-H}^-$ anions S12

S5.3 Molecular electrostatic potential analysis S13

S5.4 Localized molecular orbitals S14

S5.5 N_2 and O_2 Affinity of IPA_{-H}^- S15

References S16

S1. Methods

S1.1 Experimental methods

The infrared photodissociation (IRPD) spectroscopy experiments were conducted on the Leipzig 5 K ring-electrode ion-trap triple mass spectrometer, as described previously.[1] Deprotonated amide anions were generated and transferred to the gas phase via nanospray ion source from a mixture solution containing 0.4 mmol benzamide (Sigma Aldrich, 99%) / isophtahlamide (Tokyo Chemical Industry, Co., 97%) and 1 mmol NaF (Sigma Aldrich, 98%) in a 1:1 methanol-water mixture. The beam of anions was skimmed and subsequently thermalized to room temperature within the first He-filled radiofrequency (RF) ion guide. The CO_2-bound complexes were formed and stabilized by many collisions with the carrier gas at room temperature in the second RF ion guide, where a steady flow of pure CO_2 (Linde Gas) was introduced by a mass flow controller (MKS), as described previously.[2] The ions of interest were then mass-selected using a quadrupole mass filter and continuously trapped in a RF ring-electrode cryogenic ion trap, which was held at 14 K via a closed-cycle helium cryostat and filled with D_2 ($\approx$ 0.5 mbar). The D_2-tagged complexes were formed through three-body collisions and thermalization to the ambient temperature by collisions with background gas molecules.

All anions were extracted from the cold ion trap every 100 ms and focused both temporally and spatially into the center of the extraction region of an orthogonally mounted double-focusing reflectron time-of-flight (TOF) mass spectrometer. IRPD spectra were measured using the IR^1MS^2 scheme[3], in which the extracted anion packet was accelerated into the reflectron stage and subsequently refocused in the initial extraction region. Before reacceleration towards the MCP detector, ions with a specific mass-to-charge ratio were selectively irradiated with a properly timed, wavelength-tunable (900-3800 cm^{-1}) IR laser pulse (bandwidth: 3.5 cm^{-1}), supplied by an optical parametric oscillator/amplifier (LaserVision) laser system. IRPD spectra were recorded by continuously tuning the laser wavelength, which was monitored using a HighFinesse WS6-600 wavelength meter. The scan speed is adjusted to acquire an averaged TOF mass spectrum over 40-80 laser shots with a scan step of 2 cm^{-1}. To improve spectral quality, each spectrum was typically obtained by averaging three consecutive scans. The corresponding photodissociation cross section σ_{IRPD} was determined following the procedure described previously.[1,4]

S1.2 Theoretical methods

The initial conformational ensemble for the studied anionic species was explored with the global optimizer algorithm (GOAT) implemented in ORCA program package (version 6.0.0)[5,6], employing the semiempirical tight-binding method GFN2-xTB2 for efficient screening[7]. The geometry optimization and harmonic vibrational frequency analysis of the resulting minimum-energy structures were carried out at the B3LYP-D3BJ/aug-cc-pVTZ level of theory.[8–12] All electronic structure calculations were performed using the Gaussian 16 rev. C01 program package.[13] Simulated IR spectra were obtained by convoluting the stick spectra with a Gaussian function with FWHM = 3.5 cm^{-1} and a Lorentzian line shape function with FWHM = 8 cm^{-1}, to match the linewidths observed in the experimental spectra. Additionally, vibrational anharmonic effects on the IR spectra were evaluated using standard vibrational perturbation theory (VPT2).[14]

The accuracy of the VPT2 approach can be sensitive to the treatment of near-degeneracies. In the implementation of VPT2 incorporated in the Gaussian 16 program package, these resonances are identified through the so-called Martin test[15], which relies on an assumption that the energy differences between pairs of states is not affected by the inclusion of anharmonicity. In other words, the analysis assumes comparable anharmonic effects for all vibrations. This is often not the case, as stretching vibrations often show much more anharmonicity than bending vibrations, and the challenges increase with larger molecules due to increased density of vibrational states. Such a situation is evident in the feature of the spectrum of $[IPA_{-H}(CO_2)]^{-}\cdot D_2$ between 3100 and 3300 cm^{-1}, we have adjusted the energies and intensities that are calculated using VPT2 by including in the space of nearly degenerate states: all of the states with two quanta of excitation on the vibrations with harmonic frequencies between 1640 and 1750 cm^{-1}, which include the NH wagging and CO stretching vibrations, the state with one quantum of excitation in the NH_2 symmetric stretching vibration, and states with one quantum of excitation in the NH_2 symmetric stretch and one in a vibration with a frequency under 200 cm^{-1}. These low-frequency vibrations involve motions that break the N–H···O hydrogen bond and combination transitions involving these vibrations are expected to gain intensity due to shifts in the NH stretching frequency when this hydrogen bond is broken.[16–18] The result of this treatment is referred to as the modified VPT2 spectrum, and is shown in blue in Figure S3d.

S2. Quadrupole mass spectra

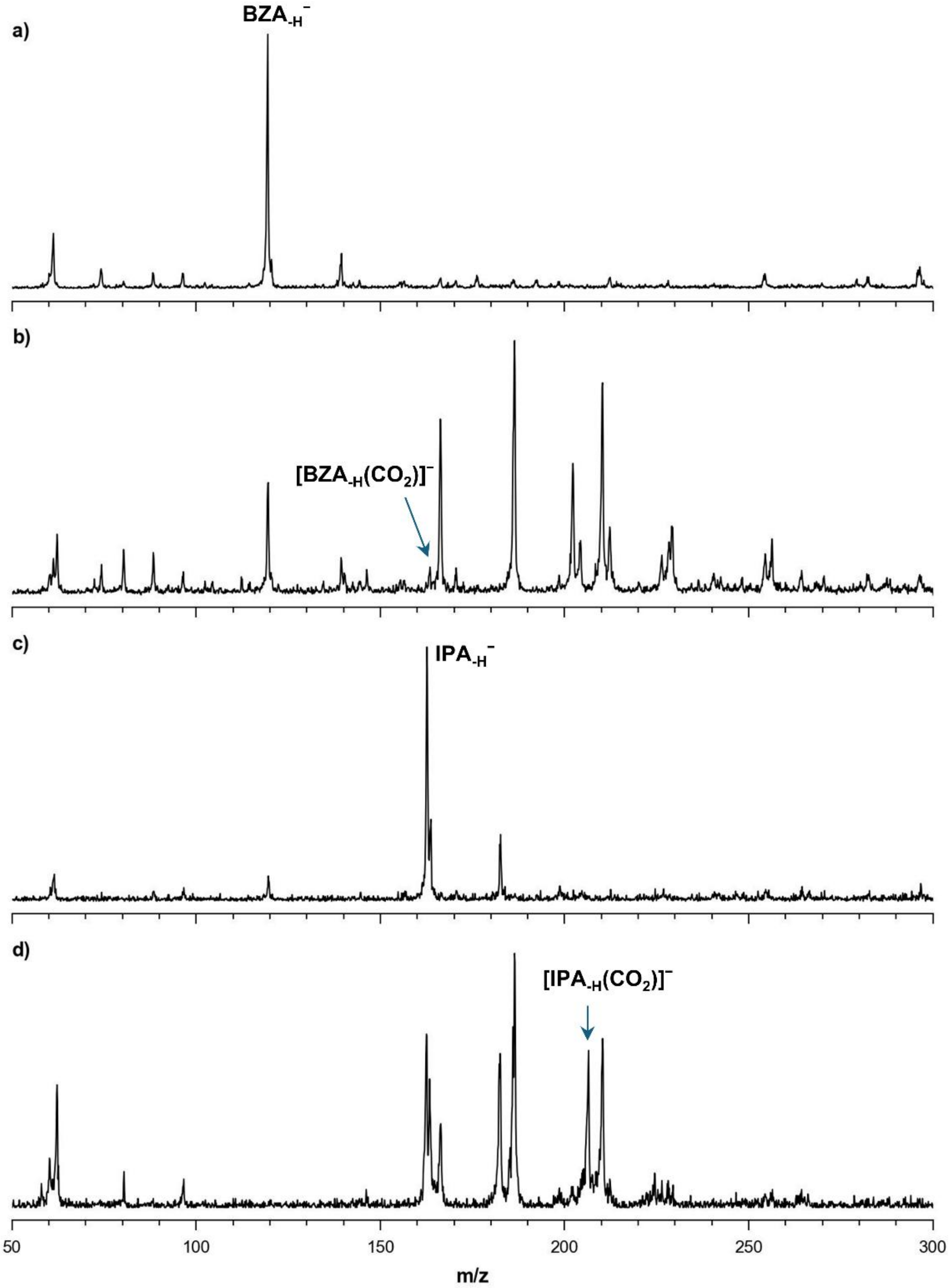

Figure S1. Mass spectra of the benzamide solution (a), the benzamide solution with CO_2 in the ion guide (b), the isophtahlamide solution (c), the isophtahlamide solution with CO_2 in the ion guide (d).

S3. Band assignments

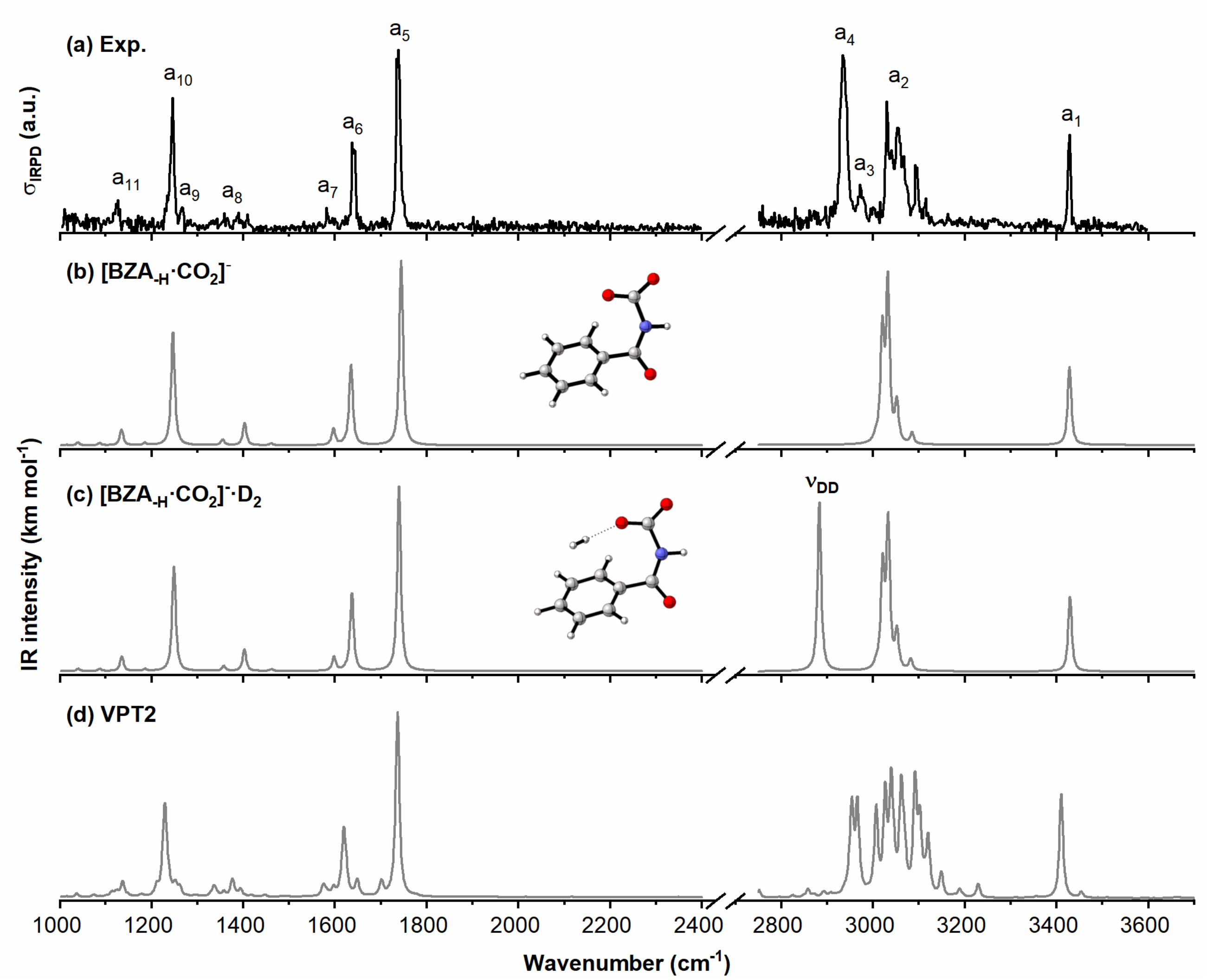

Figure S2. Comparison of the IRPD spectrum of $[BZA_{-H}\cdot CO_2]^{-}\cdot D_2$ (a) with the calculated spectra of **B1** isomer (b), and the D_2-tagged **B1** isomer (c), both scaled by 0.988 for the fingerprint region and 0.956 for mid-IR region; as well as modified VPT2 spectrum of **B1** isomer (d). All calculated spectra were obtained at the B3LYP-D3BJ/aug-cc-pVTZ level of theory. The corresponding structures are also shown.

Table S1. IRPD band positions of $[BZA_{-H}\cdot CO_2]^{-}\cdot D_2$, compared with B3LYP-D3BJ/aug-cc-pVTZ harmonic vibrational frequencies (scaled by 0.988 below 2400 cm^{-1} and 0.956 above 2400 cm^{-1}), selected VPT2 anharmonic frequencies (in cm^{-1}) with their corresponding intensities given in parenthesis (in km mol^{-1}), and band assignments.

IRPD bands		Harmonic freq.	Anharmonic freq.	Assignment[a]
a_1	3430	3417(23)		ν_{NH}
a_2	3133<br>3099<br>3057<br>3033	3075(3)<br>3042(12)<br>3023(48)<br>3011(33)		ν_{CH}
a_3	2973		2966(14)<br>2954(13)	ν_{CH}<br>$\nu_{CO_2}^{as} + \delta_{CO_2}$
a_4	2936	2874(53)		ν_{DD}
a_5	1734	1744(826)		$\nu_{CO_2}^{as}$
a_6	1640	1634(361)		$\nu_{amide\ I}$
a_7	1582	1596(73)		ν_{CC}
a_8	1388<br>1364	1403(103)<br>1355(26)		$\nu_{amide\ II}$<br>δ_{NH}
a_9	1264		1262(31)<br>1252(36)	$\delta_{CO} + \delta_{NH}$<br>$\omega_{CH} + \delta_{NH}$
a_{10}	1248	1246(509)		$\nu_{CO_2}^{ss}$
a_{11}	1128	1134(72)		δ_{CH}

[a] Labeling of vibrational modes: ν (stretching vibration), ν^{ss} (symmetric stretching vibration), ν^{as} (antisymmetric stretching vibration), δ (bending vibration), ω (wagging vibration).

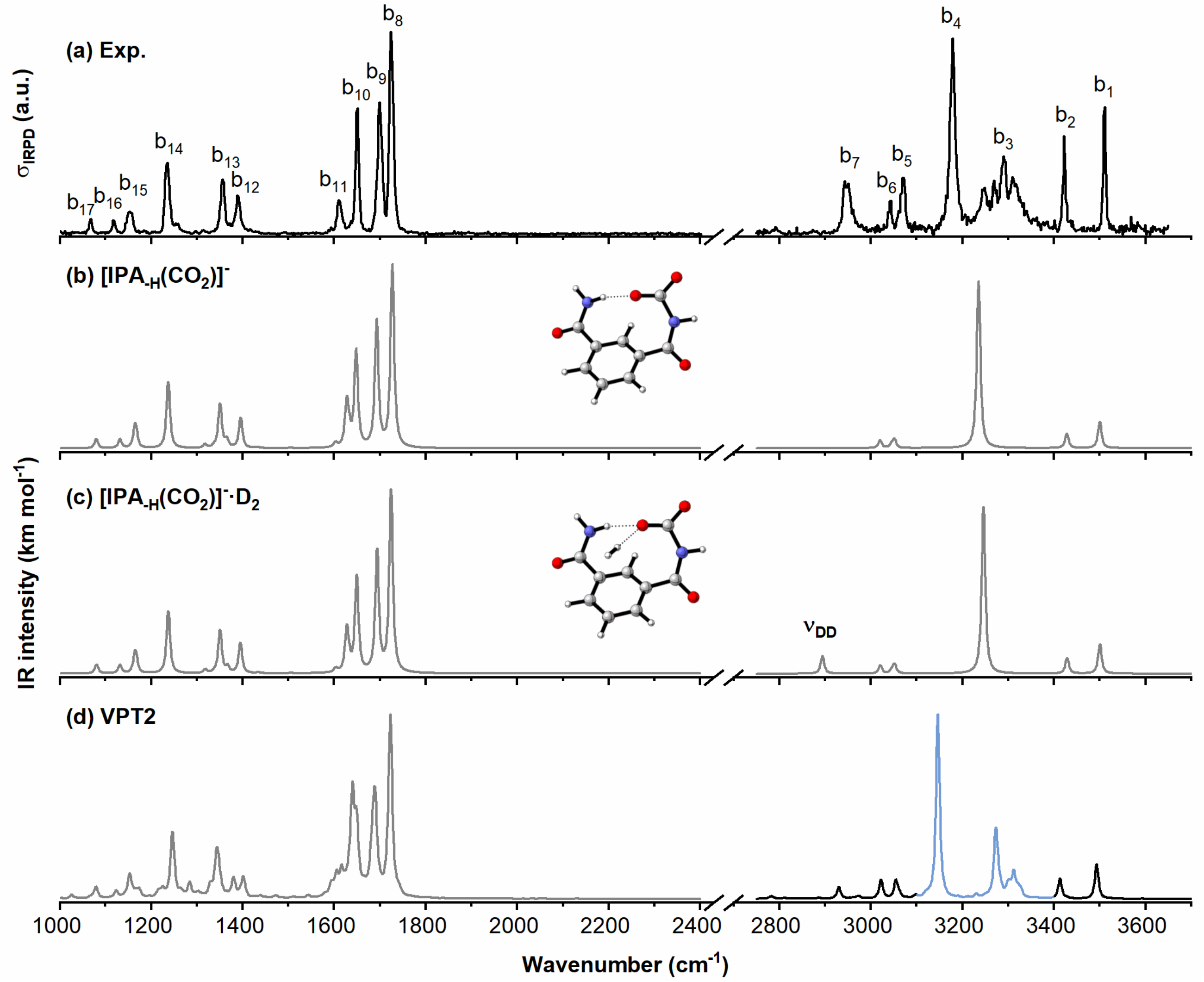

Figure S3. Comparison of the IRPD spectrum of $[IPA_{-H}(CO_2)]^{-}\cdot D_2$ (a) with the calculated spectra of **I1** isomer (b), and the D_2-tagged **I1** isomer (c), both scaled by 0.988 for the fingerprint region and 0.956 for mid-IR region; as well as modified VPT2 spectrum of **I1** isomer (d). All calculated spectra were obtained at the B3LYP-D3BJ/aug-cc-pVTZ level of theory. The corresponding structures are also shown.

Table S2. IRPD band positions of $[IPA_{-H}\cdot CO_2]^{-}\cdot D_2$, compared with B3LYP-D3BJ/aug-cc-pVTZ harmonic vibrational frequencies (scaled by 0.988 below 2400 cm^{-1} and 0.956 above 2400 cm^{-1}), selected VPT2 anharmonic frequencies (in cm^{-1}) with their corresponding intensities given in parenthesis (in km mol^{-1}), and band assignments.

IRPD bands		Harmonic freq.	Anharmonic freq.	Assignment[a]
b_1	3511	3489(54)		ν_{NH}^{free}(NH$_2$ group)
b_2	3422	3417(30)		ν_{NH}^{free}(carbamate group)
b_3	3313<br>3289<br>3270<br>3246		3312(25)<br>3298(9)<br>3273(65)<br>3231(4)	$\nu_{CO_2}^{as}+\nu_{amide\ I}$; δ_{NH_2}; ν_{NH}^{free}<br>$\nu_{NH}^{free}+\nu_r$; $\nu_{CO_2}^{as}+\delta_{NH_2}$; $\nu_{amide\ I}$<br>$\nu_{amide\ I}+\delta_{NH_2}$; ν_{NH}^{free}; $2\nu_{amide\ I}$<br>$\nu_{amide\ I}+\delta_{NH_2}$; $2\delta_{NH_2}$; $2\nu_{amide\ I}$
b_4	3178	3225(339)		ν_{NH}^{bound}
b_5	3070	3041(17), 3036(7)		ν_{CH}
b_6	3041	3010(16)		ν_{CH}
b_7	2947	2885(33)		ν_{DD}
b_8	1724	1727(791)		$\nu_{CO_2}^{as}$
b_9	1699	1693(548)		$\nu_{amide\ I}$
b_{10}	1652	1648(416)		$\nu_{amide\ I}$
b_{11}	1610	1627(183), 1632(50)		δ_{NH_2}
b_{12}	1388	1395(131)		$\nu_{amide\ II}$
b_{13}	1355	1365(34),<br>1350(192),<br>1338(5), 1318(16)		δ_{NH}<br>$\nu_{amide\ II}$<br>δ_{CH}
b_{14}	1236	1237(287)		$\nu_{CO_2}^{ss}$
b_{15}	1152	1166(63), 1164(55)		δ_{CH}
b_{16}	1119	1132(40)		ω_{NH_2}
b_{17}	1067	1080(40)		δ_{CH}

[a] Labeling of vibrational modes: ν (stretching vibration), ν^{ss} (symmetric stretching vibration), ν^{as} (antisymmetric stretching vibration), δ (bending vibration), ω (wagging vibration).

S4. Calculated structures

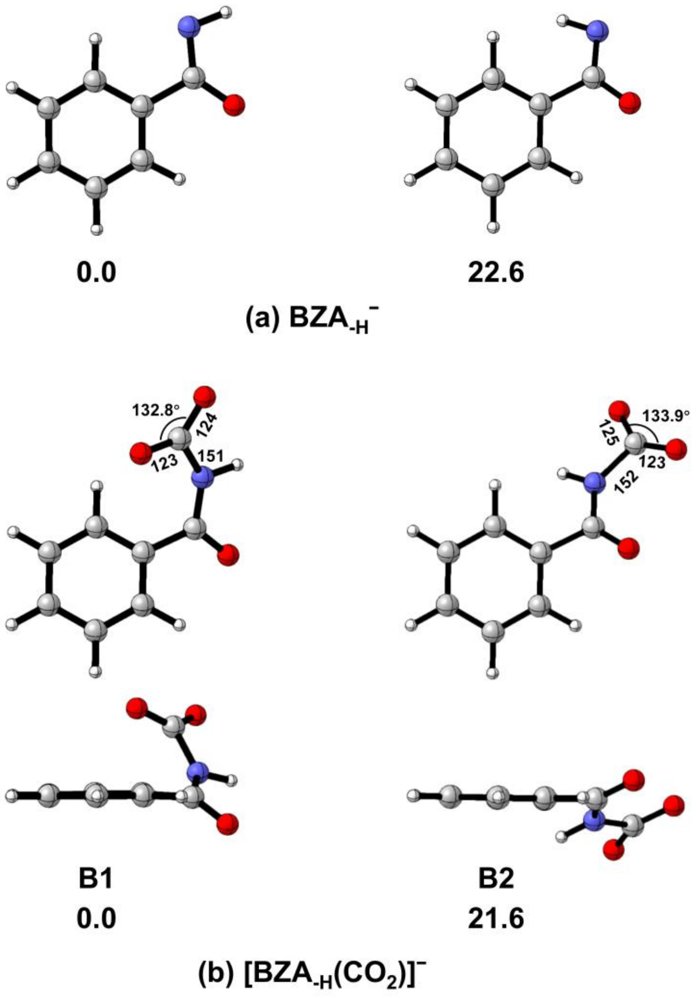

Figure S4. Molecular structures of the BZA_{-H}^{-} anion (a) and the complex of BZA_{-H}^{-} with CO_2 (b), optimized at the B3LYP-D3BJ/aug-cc-pVTZ level of theory. Relative energies with respect to the respective lowest-energy structure are given in kJ mol^{-1}. Bond lengths are listed in pm. Color code: C (grey), H (white), N (blue), O (red).

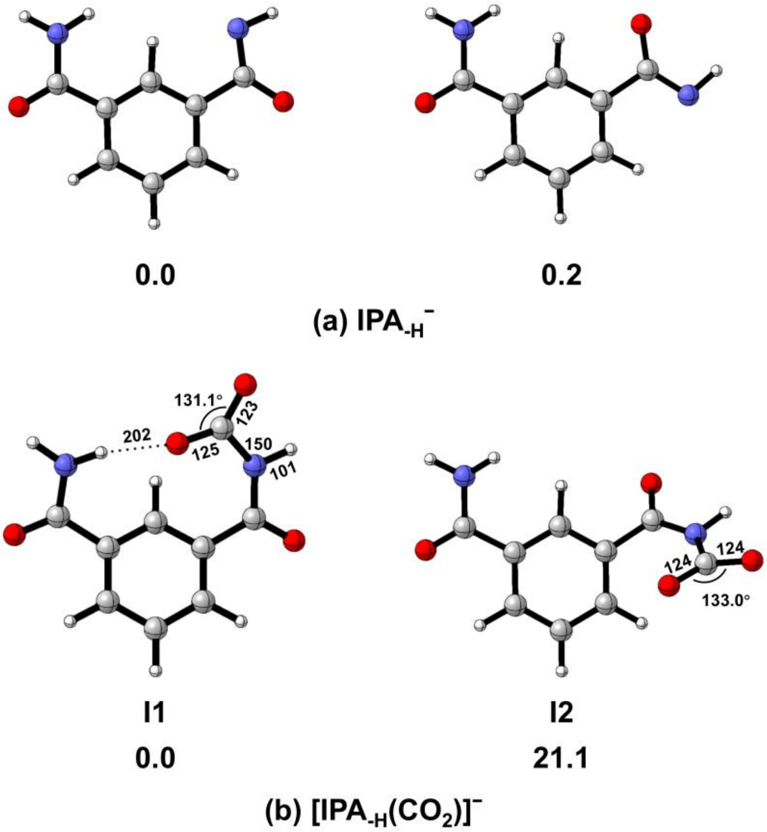

Figure S5. Molecular structures of the IPA_{-H}^{-} anion (a) and the complex of IPA_{-H}^{-} with CO_2 (b), optimized using B3LYP-D3BJ/aug-cc-pVTZ method. Relative energies with respect to the respective lowest-energy structure are given in kJ mol^{-1}. Bond lengths are listed in pm. Color code: C (grey), H (white), N (blue), O (red).

S5. Other aspects of CO_2 binding in amide vs amine carbamates

S5.1 Reaction mechanisms of amine systems with CO_2

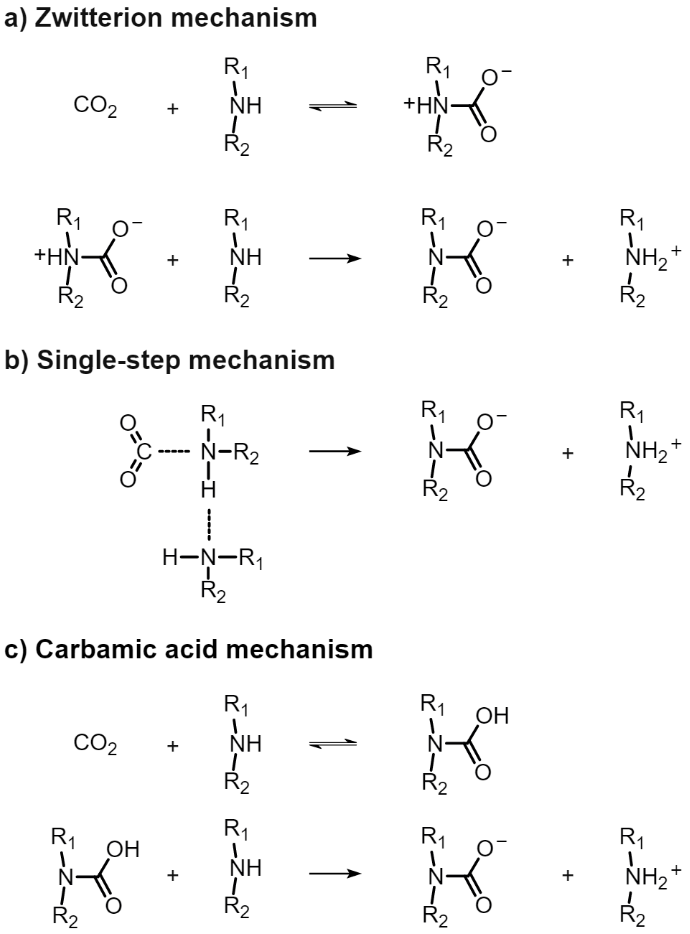

Scheme S1. Three reaction mechanisms proposed for the systems of primary and secondary amines with CO_2.

S5.2 Proton affinities of F^-, BZA_{-H}^-, IPA_{-H}^-, and $BZAN_{-H}^-$ anions

Table S3. Proton affinity values calculated for F^-, BZA_{-H}^-, and IPA_{-H}^- and deprotonated Benzylamine ($BZAN_{-H}^-$) anions at the B3LYP-D3BJ/aug-cc-pVTZ level of theory.

	**Proton affinity** (B3LYP-D3BJ/aug-cc-pVTZ, kJ mol^{-1})
**F^-**	1540
**BZA_{-H}^-**	1488
**IPA_{-H}^-**	1460
**$BZAN_{-H}^-$**	1631

S5.3 Molecular electrostatic potential analysis

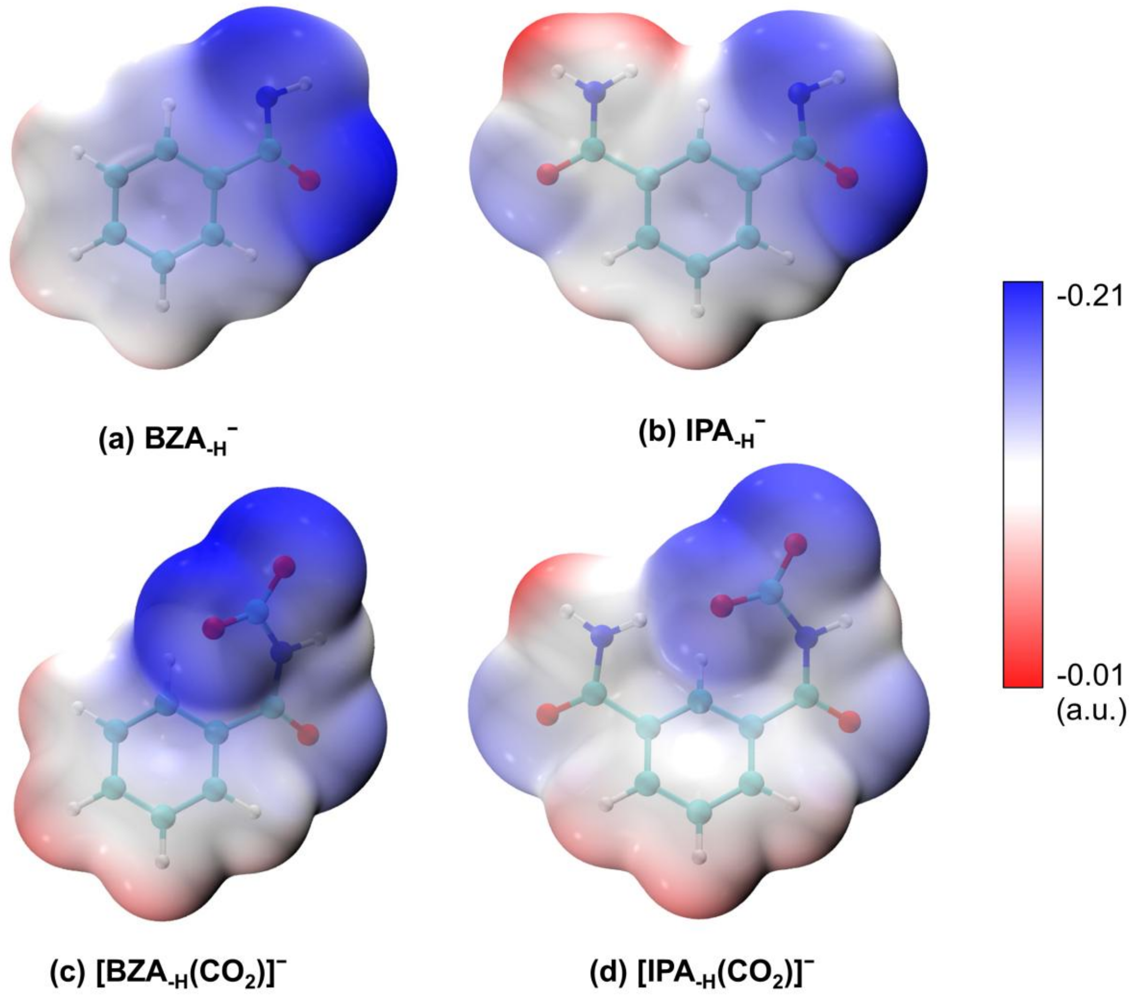

Figure S6. Molecular electrostatic potential (MESP) mapped on the 0.01 au electron density surface for the lowest-energy structures of BZA_{-H}^{-} (a), IPA_{-H}^{-} (b), $[BZA_{-H}(CO_2)]^{-}$ (c) and $[IPA_{-H}(CO_2)]^{-}$ (d). Blue regions indicate electron-rich areas while the red region corresponds to electron-deficient areas.

S5.4 Localized molecular orbitals

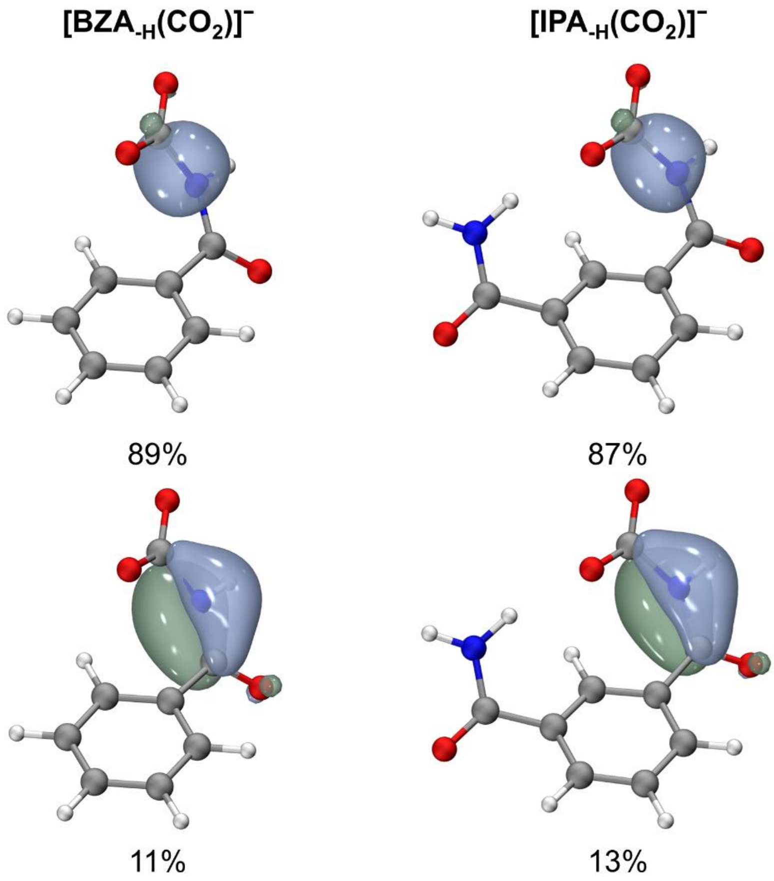

Figure S7. The localized molecular orbitals (LMOs) with the highest contribution to the N–C Mayer bond order in the **B1** and **I1** complexes, with the percentage contributions indicated below each orbital.

S5.5 N_2 and O_2 Affinity of IPA$_{-H}^{-}$

$[IPA_{-H}(N_2)]^-$

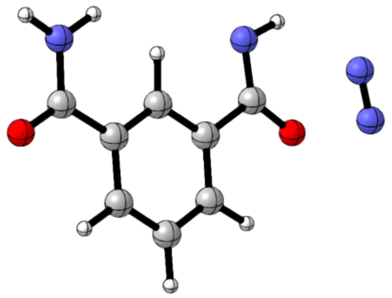

ΔE	-10 kJ mol^{-1}
ΔH	- 8 kJ mol^{-1}

(a)

$[IPA_{-H}(O_2)]^-$

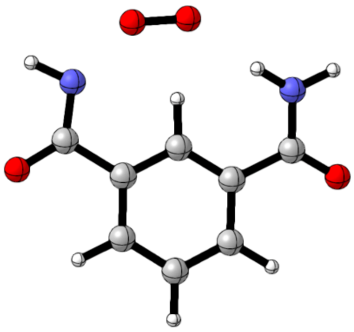

ΔE	-13 kJ mol^{-1}
ΔH	-12 kJ mol^{-1}

(b)

Figure S8. Optimized structures of the $[IPA_{-H}(N_2)]^-$ (a) and $[IPA_{-H}(O_2)]^-$ (b) anion complexes, along with their binding energies and enthalpies at 298 K calculated at the B3LYP-D3BJ/aug-cc-pVTZ level of theory.

References

[1] N. Heine, K. R. Asmis, *Int. Rev. Phys. Chem.* **2015**, *34*, 1.

[2] S. Schmahl, F. Horn, J. Jin, H. Westphal, D. Belder, K. R. Asmis, *ChemPhysChem* **2024**, *25*, e202300975.

[3] M. Mayer, K. R. Asmis, *J. Phys. Chem. A* **2021**, *125*, 2801.

[4] N. Heine, K. R. Asmis, *Int. Rev. Phys. Chem.* **2016**, *35*, 507.

[5] F. Neese, *Wiley Interdiscip. Rev.:Comput. Mol. Sci.* **2012**, *2*, 73.

[6] F. Neese, F. Wennmohs, U. Becker, C. Riplinger, *J. Chem. Phys.* **2020**, *152*, 224108.

[7] C. Bannwarth, S. Ehlert, S. Grimme, *J. Chem. Theory Comput.* **2019**, *15*, 1652.

[8] R. A. Kendall, T. H. Dunning, R. J. Harrison, *J. Chem. Phys.* **1992**, *96*, 6796.

[9] S. Grimme, J. Antony, S. Ehrlich, H. Krieg, *J. Chem. Phys.* **2010**, *132*, 154104.

[10] S. Grimme, S. Ehrlich, L. Goerigk, *J. Comput. Chem.* **2011**, *32*, 1456.

[11] C. Lee, W. Yang, R. G. Parr, *Phys. Rev. B* **1988**, *37*, 785.

[12] A. D. Becke, *J. Chem. Phys.* **1993**, *98*, 5648.

[13] M. J. Frisch, G. W. Trucks, H. B. Schlegel, G. E. Scuseria, M. A. Robb, J. R. Cheeseman, G. Scalmani, V. Barone, G. A. Petersson, H. Nakatsuji et al., *Gaussian 16 Rev. C.01*, Wallingford, CT, **2016**.

[14] V. Barone, *J. Chem. Phys.* **2005**, *122*, 14108.

[15] J. M. L. Martin, T. J. Lee, P. R. Taylor, J.-P. François, *J. Chem. Phys.* **1995**, *103*, 2589.

[16] E. M. Myshakin, K. D. Jordan, E. L. Sibert, M. A. Johnson, *J. Chem. Phys.* **2003**, *119*, 10138.

[17] N. Heine, E. G. Kratz, R. Bergmann, D. P. Schofield, K. R. Asmis, K. D. Jordan, A. B. McCoy, *J. Phys. Chem. A* **2014**, *118*, 8188.

[18] S. M. Craig, F. S. Menges, C. H. Duong, J. K. Denton, L. R. Madison, A. B. McCoy, M. A. Johnson, *Proc. Natl. Acad. Sci. U. S. A.* **2017**, *114*, E4706-E4713.
